# Factors influencing deprescribing for residents in Advanced Care Facilities: insights from General Practitioners in Australia and Sweden

**DOI:** 10.1186/s12875-016-0551-7

**Published:** 2016-11-05

**Authors:** Beata Borgström Bolmsjö, Anna Palagyi, Lisa Keay, Jan Potter, Richard I. Lindley

**Affiliations:** 1 Department for Clinical Sciences, Lund University, Malmö, Sweden; 2 The George Institute for Global Health, Sydney Medical School, University of Sydney, Sydney, NSW Australia; 3 Illawarra Health and Medical Research Institute, University of Wollongong, Wollongong, NSW Australia

**Keywords:** Deprescribing practice, General Practitioners, Polypharmacy, Ageing, Advanced Care Facilities, Nursing homes

## Abstract

**Background:**

General Practitioners (GPs) are responsible for primary prescribing decisions in most settings. Elderly patients living in Advanced Care Facilities (ACFs) often have significant co-morbidities to consider when selecting an appropriate drug therapy. Careful assessment is required when considering appropriate medication use in frail older patients as they have multiple diseases and thus multiple medication. Many physicians seem reluctant to discontinue other physicians’ prescriptions, resulting in further polypharmacy. Therefore it is relevant to ascertain and synthesise the GP views from multiple settings to understand the processes that might promote appropriate deprescribing medications in the elderly.

The aims of this study were to 1) compare and contrast behavioural factors influencing the deprescribing practices of GPs providing care for ACF residents in two separate countries, 2) review health policy and ACF systems in each setting for their potential impact on the prescribing of medications for an older person in residential care of the elderly, and 3) based on these findings, provide recommendations for future ACF deprescribing initiatives.

**Methods:**

A review and critical synthesis of qualitative data from two interview studies of knowledge, attitudes, and behavioural practices held by GPs towards medication management and deprescribing for residents of ACFs in Australia and Sweden was conducted.

A review of policies and health care infrastructure was also carried out to describe the system of residential aged care in the both countries.

**Results:**

Our study has identified that deprescribing by GPs in ACFs is a complex process and that there are numerous barriers to medication reduction for aged care residents in both countries, both with similarities and differences. The factors affecting deprescribing behaviour were identified and divided into: intentions, skills and abilities and environmental factors.

**Conclusions:**

In this study we show that the GPs’ behaviour of deprescribing in two different countries is much dependent on the larger health care system. There is a need for more education to both GPs and ACF staff as well as better cooperation between the different health care systems and appropriate monetary incentives for elderly care to achieve better conditions for deprescribing practice.

## Background

Polypharmacy is common in frail elderly patients requiring residential care, and is associated with an increased risk of serious adverse drug effects and hospitalisation [1, 2]. Studies in aged care facilities (ACFs) show that polypharmacy (defined as the concomitant use of five or more medicine) occurs in over 90 % of residents [3, 4], with an average of 7–10 medications per person [5]. Further, over 70 % of ACF residents take one or more potentially inappropriate medication (PIM) [5] − medicines associated with higher medical costs, increased rates of adverse drug effects and poorer health outcomes.

Frailty is a construct originally established by gerontologists to describe cumulative declines across different physiological systems that occur with ageing, leading to a state of diminished physiological reserve and increased vulnerability to stressors [6]. Different approaches to frailty exist and different screening criteria for frailty as a syndrome have been developed. An example of screening for frailty by Fried et al. requires the presence of a critical mass (≥3) of the following clinical manifestations: weakness, weight loss, slow walking speed, fatigue, and low levels of activity [7]. This phenotype has been found to predict various poor clinical outcomes, including falls, the development of disability, hospitalization, and mortality.

The term deprescribing is used to describe the process of gradual withdrawal and ceasing of PIMs, supervised by a health care professional and with the goal of managing polypharmacy and improving outcomes [8]. Deprescribing initiatives in ACFs are not well explored and intervention studies assessing the effectiveness of deprescribing interventions on health, quality of life and mortality in older people residing in aged care remain in their infancy [9] However these studies have struggled with the inclusion of patients, and therefore there is a need to explore attitudes about deprescribing in different ACF settings.

General Practitioners (GPs) are responsible for primary prescribing decisions in most settings. Elderly patients often have significant co-morbidities to consider when selecting an appropriate drug therapy. Careful assessment is required when considering appropriate medication use in frail older patients as they have multiple diseases and thus multiple medication. There is sometimes a perceived lack of communication between GPs and other specialists concerning their patients’ medication, and this might reduce treatment quality [10]. Many physicians seem reluctant to discontinue other physicians’ prescriptions, resulting in further polypharmacy, and other health care professionals address this question as beyond their control [11]. Therefore it is relevant to ascertain and synthesise the GP views from multiple settings to understand the processes that might promote appropriate deprescribing.

Even though the systems of elderly care differ between countries, there is a major need for in-depth research on the work and quality of care in ACFs to recognize opportunities for strategic improvement and to highlight priorities for education [12], which can be used for deprescribing practice.

The aims of this study were to 1) compare and contrast behavioural factors influencing the deprescribing practices of GPs providing care for ACF residents in two separate countries, 2) review health policy and ACF systems in each setting for their potential impact on the prescribing of medications for an older person in residential care of the elderly, and 3) based on these findings, provide recommendations for future ACF deprescribing initiatives.

## Methods

A review and critical synthesis of qualitative data from two studies of knowledge, attitudes, and behavioural practices held by GPs towards medication management and deprescribing for residents of ACFs in Australia and Sweden was conducted. A qualitative approach was addressed as the point of departure consists of concrete descriptions of experienced events from the perspective of everyday life by the participants. The result is then a description of underlying comprehension of the experience, interpreted for assessing knowledge that could be significant for a wider community [13].

The methods for each study have been published elsewhere [14, 15]. In brief, Study 1 (The RELEASE Study) incorporated a focus group of eight GPs, all of who provided regular medical care to residents of ACFs in a single region in south-eastern Australia. Study 2 (The Swedish GP Study) involved individual semi-structured interviews with 12 GPs working in primary health care centers throughout the southernmost province of Skåne, Sweden. In both of the studies the GPs were recruited by email or newsletter announcements. The inclusion criterion for the participating GPs was that he or she had responsibility for patients at an ACF. The interviewed GPs did not get any reward for participating in the study, as it was based on voluntariness. The recruitment of GPs as well as the interviews and the focus group discussion with the GPs lasted until the researchers felt that sampling more data would not lead to more information related to the research question i.e. saturation was reached.

A review of policies and health care infrastructure was carried out to describe the system of residential aged care in the both countries. The search was conducted on the internet for government documents concerning the following areas: demographics of the aged population, proportion of population in ACFs, number of medications per ACF resident, primary health care structure, ACF GP workforce, funding for primary health care, GP funding structure, and medication reviews for ACF residents

### Data analysis

**Fig. 1:**
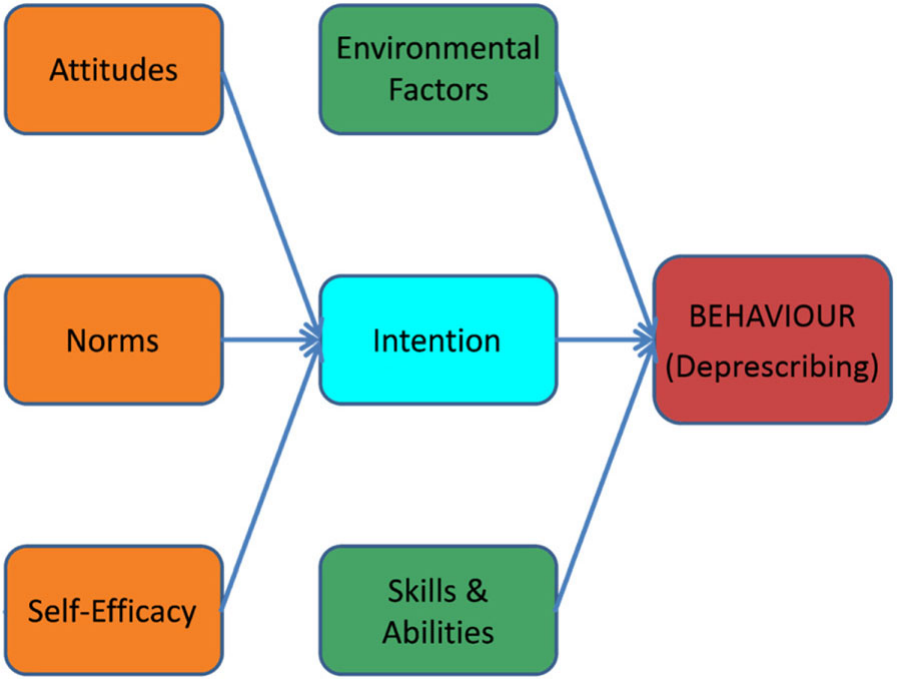
An Integrative Model of Behaviour Prediction, adapted from Fishbein (2006)

The Integrative Model of Behaviour Prediction (IMBP) [16] was chosen as the theoretical framework underpinning the interpretation of findings from both studies. The IMBP is a theoretical model used widely in health behaviour research which predicts that intentions are the immediate antecedents of behaviour, and recognizes that environmental factors, skills and abilities can moderate the intention-behaviour relationship. The model also assumes that the intention to perform a specified behaviour is a function of attitudes, perceived normative pressure and self-efficacy [17] (Fig. 1).

The verbatim transcripts from the Australian GP focus group and the Swedish GP interviews were read through several times by the research team for content emersion. All researchers read the full verbatim transcripts from the Australian study. The Swedish study was transcribed verbatim in Swedish, why only the first author who is Swedish-speaking could understand the transcripts and then translate them into English for the rest of the research team to read. Findings across each study were then explored by all members of the research team, coded and systematically collapsed under each of the key constructs of the IMBP, focusing on the constructs of attitudes, norms, self-efficacy, environmental factors, intent, and skills and abilities. Themes additional to those falling within the IMBP structure (if any) were allowed to emerge inductively. The research team worked collaboratively to further refine themes and compare findings according to each study.

## Results

### Aged care and primary health care in Australia and Sweden

A comparison of aged care systems and policies in Australia and Sweden is presented in Table 1.

**Table 1:** Aged care systems and policies in Australia and Sweden

	Australia	Sweden
Population ≥65 years	15 % [21]	20 % [22]
Population ≥65 in ACF	7.8 % [23]	5 % [22]
Number of medications per ACF resident	7–10 [3, 4, 24]	7–10 [25, 26] (>70 % have one or more PIM)
ACF providers	• Private not-for-profit [27] • Private for-profit [27]	• Municipality (responsible) • Private (paid by municipality) [28]
General practice structure	• Single/multiple GP private practices [29] • Small − medium business model [29]	• Team-based primary care facilities • Most public (owned by the county councils) • Few private (mostly owned by companies or cooperatives) [30]
GPs in ACF	• Continuity model: GP follows long-term patient to ACF [31] • ACF panel model: GP provides care for >2 patients in nearby ACF [31] • GPwSI ACF model: GP provides regular scheduled service to large number of ACF residents [31] • LGPT model: GP part of team-based care [31] • ACF-based model: Single GP partners with single ACF [31]	• County councils responsible for residents’ medical care; generally weekly visit to ACF by one GP from the local primary care unit [28]
Funding for primary health care	• Government funded (both state-, territory- and local-) • Fee-for-service paid directly by patients and clients • Private health insurers • Private charities [32]	• Funded through national and local taxation [30].
GP funding structure	• Fee-for-service paid directly by patients, and/or • Reimbursement by Government Medicare Benefits Schedule [29]	Different funding in the 20 different county councils [33]. In Skåne where interviewed GPs worked [34]: • Based on capitation for registered patients. • Complemented with estimated ‘illness burden’ indexation • Performance-based payments
Medication reviews for ACF residents	• Pharmaceutical review outsourced by ACF to private company or local pharmacist. • Compulsory biennially as part of ACF accreditation process [35]. • GP may request local pharmacist to undertake medication review at any time [35]. • Funded by Government Medicare Benefits Schedule; maximum 1 review in 12 month period.	• Undertaken by county council employed pharmacists undertaken at any time, at least once a year, aiming to increase quality of medications and reduce PIMS [36].

*ACF* aged care facility, *GP* general practitioner, *GPwSI ACF* GPs with special interest in aged care facilities, *LGPT* longitudinal general practice team

In Sweden the medical care of ACF residents is managed by the local primary health care (PHC) centre. A single salaried GP is allocated to one ACF, provides weekly visits to residents, and prioritises care in consultation with the ACF Registered Nurse (RN). In the last decade, intervention studies with patient focused drug surveillance in ACFs in Sweden have showed a reduction of the number of prescription medications per resident [18]. Structured medication reviews are conducted regularly by pharmacists in collaboration with the GP, the RN and ACF nursing staff, and have been shown to improve the appropriateness of prescribing, including the deprescribing of unnecessary medications for older residents with multiple diseases [19]. To incentivise the reduction of PIMs, the Swedish government provides a financial reward to regions that reduce PIM rates by a given percent [20]. In the southernmost province of Skåne (where the interviewed GPs are situated) there is an appointed “Elderly-General”, a GP with special interest in aged care and medications who assists with medication reviews and educates other GPs on deprescribing practice.

In Australia, there are a number of different models of GP care for residents of ACFs (http://www.racgp.org.au/afp/2015/april/models-of-general-practitioner-services-in-residential-aged-care-facilities/). A fall in the number of GP consultations for residents of ACFs has been reported and several barriers to the provision of GP services to ACFs have been identified, including competing with the complexity and demands of regular general practice commitments, the time consuming nature of visits to ACF residents, and inadequate remuneration for the work conducted and the travel time [21]. Initiatives to increase GP services for ACF residents include improved reimbursement, improved collaborative care initiatives involving allied health, and an enhanced role in palliative care. Additional research is needed to investigate the potential impact of innovative models of care and alternative funding methods in Australia [22].

### Intention to deprescribe

According to the IMBP (Fig. 1) [17], the intention for a GP to perform deprescribing is formed by critical beliefs in 1. self-efficacy, 2. norms and 3. attitudes. The themes emerging from the transcripts regarding these beliefs are outlined below and further summarised in Table 2.

**Table 2:** Beliefs forming a General Practitioner’s intention to deprescribe

1. Self-efficacy	I deprescribe	*“I cease Warfarin [for] all my nursing home patients without exception because I think it’s actively dangerous to be on Warfarin.” (AusGP4)* *“As soon as a problem arises, I take a look at the medication list and figure out which one to deprescribe.” (SweGP10)*
	Insecurity	*“Say someone was [on] Parkinsonism drugs - I would be less confident stopping it because… I do initiate anti-Parkinsonism drugs, but not at the higher end of them.” (AusGP6)* *“And where it can be hard to gain support for examinations and follow-ups and help with observations and so… they like to call for sedatives, when instead there is a need of attendance and measures other than medications.” (SweGP10)*
	Evidence and know-how	*“From a University point of view, if you could train the undergraduate to be interested in coming to the nursing home. This is the greatest point…” (AusGP8)* *“I have only had one course on elderly and medications, and that was long ago. But I still use the notes from that class.” (SweGP1)* *“Is it right or wrong to deprescribe this medication? You are pretty alone in the decision actually. I would like some kind of mentorship or someone to talk to.” (SweGP3)*
2. Norms	Unrealistic expectations	*“And, I think, sometimes the specialists are a bit unrealistic. Sometimes they’re a little bit unrealistic about what’s actually going on - on the actual coalface, I think.” (AusGP4)* *“I don’t think they need some of the medications, but it is all psychology, the psychology of the patient and of the staff. They believe somehow that somethings would get better with pills.” (SweGP5)*
	The Almighty doctor	“*So they’re [relative] feeling guilty about the fact that they’ve [their parent] gone into the nursing home…So the…family want them to keep on going and going and going, so you do everything possible to keep them [resident] alive.” (AusGP6)* *“There is a focus on the doctor. And I have very little chance to help the patient because what the patient actually is in need of is basic care …but this may lead to that a patient gets many medications” (SweGP11)*
3. Attitudes	Facilitating a good quality of life	*“I think the medications which keep them comfortable are important, like pain medications can help them. And those ones which are related to heart.” (AusGP5)* *“…the first priority is definitely to reduce suffering, reduce anxiety…try to make life meaningful for the patient. Diseases are secondary.” (SweGP4)*
	Interest and disinterest in aged care	*“I think that’s a big barrier for us to be able to get other doctors [to] actually provide services there [in the aged care facility].” (AusGP6)* *“It [aged care work] is sort of a relief compared to the ordinary work at the primary health care centre, you get away from the primary health care centre for a while every week and it is freer time, not the scheduled appointments all the time, but you go there and sometimes you visit the patients and sometimes you just discuss the patients. It is more free and a different way of working with patients.” (SweGP12)*

#### Self-efficacy in deprescribing

Self-efficacy is based on a GP’s beliefs that they are able to carry out the processes required to deprescribe.

##### I deprescribe

Both the Australian and Swedish GPs expressed confidence in their ability to deprescribe.

*“After 20 years I know exactly what I want to do. I don’t have a problem with saying, yep, the Statin goes, the Aspirin goes, the Warfarin goes.” (AusGP4)*

*“When a patient first arrives to the nursing home, it is no longer ‘what to prescribe?’, but ‘what to deprescribe?’” (SweGP5)*

##### Insecurity

In cases where the GP felt that a resident’s medication could be replaced by nursing care, their self-efficacy to deprescribe was lower as they perceived a lack of support from ACF nursing staff in “prescribing” nursing care when deprescribing medications

*“I am pretty confident in what I am doing and what I don’t want to do. If there is a prescription that I don’t agree with, I won’t prescribe it…But when it comes to the discussion about non-pharmacological issues, such as nursing care instead of medications, it is hard to gain support from the nursing staff.” (SweGP6)*

Common to GPs from both settings was a lack of confidence to deprescribe disease-specific medications without the input of specialist advice. In these cases the GPs felt isolated in their medication decision making, as consulting a specialist was often difficult to initiate and time consuming.

*“They seem to have a pretty crappy end stage level of Parkinson’s, but maybe it would be worse if I stopped these tablets and so I’ll get anxious about that.” (AusGP6)*

*“Sometimes I try to call a geriatrician, but there is no easy way to contact them. It all ends up with me writing a letter to try to get a hold of someone, because via telephone it is just hopeless.” (SweGP9)*

##### Evidence and know-how

There were concerns in both settings over skill development and availability of information to allow evidence-based deprescribing. The GPs in Sweden felt that education for deprescribing practice was lacking and identified the need for a forum for meeting other GPs in elderly care for discussions about deprescribing. Australian GPs expressed worry about not knowing enough facts for deprescribing and emphasized the importance of educating the next generation GPs in the care of older patients.

“*I’m not clever enough to have all the statistics in my head to be able to say, well, that Statin is stopping all that absolute relative blah de blah, which I don’t understand very well. So I can’t really educate the patient off the top of my head.” (AusGP4)*

*“It would be a great help [with deprescribing] to have further training and to meet with GPs in the same situation.” (SweGP6)*

Residents of ACFs in both countries had regular medication reviews conducted by a pharmacist and this was seen as a facilitator of deprescribing by the Swedish GPs:

*“I think that the quality [of prescribing decisions] has increased greatly, and that is actually because of Hälsovalet [government incentives of medication reviews]… It makes my work with the elderly more pleasant.” (SweGP3)*

Australian GPs, however, were less inclined to implement the recommendations of a medication review conducted by a pharmacist from external medication review companies, who were often contracted by ACFs to conduct their biennial accreditation medication review:

*“It’s one of those pieces of paper which goes in the shredder for me. If I haven’t got the time to negotiate – or to really think about the [medication review] because medico legally it’s a document which actually stitches you up.” (AusGP6)*

#### Norms affecting GP-led deprescribing

The expectations of other stakeholder involved in the care of a resident, including RNs, nursing staff and relatives of residents, are often based on perceived norms and are important influencers on a GP’s overall intent to deprescribe.

##### Unrealistic expectations

Both the Swedish and Australian GPs thought that relatives, nursing staff and residents had unrealistic views on the role and importance of medications for an older person. Australian doctors expressed a gap between relatives’ expectations of care provided by an ACF, and suggested a need for discussion and acceptance of a palliative approach to the care of ACF residents.

*“In our world there is a lot of fixation on diagnoses and diseases that should be cured. But in residential aged care there should be another perspective – having company, not being alone etc.” (SweGP4)*

*“I honestly think 90 % of relatives don’t see the nursing homes as a palliative situation… Well, people with medical training can, perhaps, see it for what it is. Whereas relatives look at things often through rose-coloured glasses and they think it’s going to get better.” (AusGP1)*

##### The Almighty doctor

GPs from both settings perceived pressure from relatives and nursing staff to prescribe medications in order to try to improve a resident’s health state, and felt this was a barrier to their attempts to deprescribe.

*“So the anxiety that the staff [and] a lot of the relatives have about grandma dying, they’re displaced onto the nursing staff, so the nursing staff are worried about grandma and they’ve displaced it onto us and then that gets displaced onto… the geriatrician” (AusGP6)*

*“I want to get away from giving medicines when the reason is that there are not enough nursing staff. There is a tendency for this, with too much focus on the doctor.” (SweGP11)*

#### Attitudes towards deprescribing

##### Facilitating a good quality of life

Swedish GPs perceived their role in the care of a resident as being a facilitator of good quality, end of life care. Deprescribing was seen as an important component of this approach.

*“I want to give the patients a good quality of life, and I follow them in the continuum of ageing, with their progressive weakness and adapt medical interventions for this.” (SweGP10)*

The attitudes of the Swedish GPs seemed to be based on their own perspectives of ageing and dependency and how they would like to be treated in that stage of life.

*“Every time I am there I think to myself, ‘please don’t let me end up here’.” (SweGP5)*

*“I have done much thinking about how I want to be treated in that situation, and I have talked to my relatives about it. If I get seriously ill and suffer from dementia, please don’t fight for prolonging my life to eternity” (SweGP2)*

While there was some sentiment from Australian GPs about a desire to assist residents to achieve the best quality of life possible, this was often overshadowed by discussion of perceived system-related barriers to providing care.

*“So [the aim is to] to treat them, to keep them comfortable. And, again, those who have a good quality life, they can go out and visit their relatives, that is just like normal management.” (AusGP5)*

##### Interest and disinterest in aged care

Interest in providing care for ACF residents differed notably between the GPs from the two countries. The Australian GPs were overwhelmingly negative about aged care and expressed dissatisfaction at the financial reimbursement provided for ACF services. Their attitudes towards deprescribing for ACF residents were influenced by concerns of blame in the case of negative health outcomes, an increasing workload and a general disinterest in aged care.

*“I’d be very, very happy to give that to anybody who wanted it, pay a significant amount of money to get that off my hands. I’m completely burnt out with it.” (AusGP4)*

*“In my heart I know it would have made no difference, but I’ve had it where people – I’ve stopped the bisphosphonate or whatever and within the week they’ve fallen over and busted something. And the nurses… they go, ‘oh, look’.” (AusGP6)*

The Swedish GPs expressed a more positive attitude about working in ACFs and often preferred it to their more stressful everyday work at the PHC centre.

*“It is like a relief compared to the PHC centre, that you get to go away from the PHC centre every week and go to the nursing home, and it also gives you an opportunity to prioritise your work.” (SweGP12)*

##### Behaviour of deprescribing

In addition to a GP’s intention to deprescribe, the IMBP (Fig. 1) denotes that both environmental factors and skills and abilities will influence whether or not this behaviour is performed. The themes emerging from the transcripts that relate to these two constructs are outlined below and expanded in Table 3.

**Table 3:** Factors affecting a General Practitioner’s deprescribing behaviour

Environmental factors	Working within a complex system	*“So lack of uniformity of medication documentation is a barrier.” (AusGP3)* *“I am pretty stressed when I am come back to the PHCC from the ACF. It limits me to not have the computer system to work with at the ACF, therefore I have to bring back a lot of work to administrate when I come back to the PHCC.” (SweGP6)*
	Communication	*“You’re stopping people’s blood pressure medication and then to Digoxin and things like that. To me what that means is then unless you then identify a palliative care situation and everybody’s happy with that, is that means more monitoring, more faxes, more this, more that.” (AusGP3)* *“I feel that it takes a lot of patience and ability to cooperate with the other staff at the ACF.” (SweGP2)*
	Financial incentives to providing care	*“They will all complain bitterly about the doctors because we’re always - well, I am - always grumpy and never want to be there because you feel like you’re virtually doing charity work because you work hard.” (AusGP4)*
Skills and ability	Quality of human resources	*“The issue is as well is that the nursing staff have got to have the capacity to actually adhere to the plan.” (AusGP4)* *“I would like more nursing staff, better educated nursing staff. It is my belief that we could save a lot of time and money that way.” (SweGP2)*
	Quantity of human resources	*“The institution has to respect the [prescribing] policy and have enough skill to actually adhere to it.” (AusGP6)* “*It is not optimal that a patient gets a sedative drug instead of someone that holds her hand, but it is as good as it can get because there is no other way. That is frustrating of course, and sad, that I can’t influence this in any way” (SweGP2)*

#### Environmental factors influencing deprescribing

##### Working within a complex system

The administrative load associated with ACF work was burdensome for the GPs in both countries. Messy medication charts and poorly integrated IT systems were seen by GPs as barriers to efficient medical care. The Swedish GPs also highlighted the onerous administrative situation with different employers, as the ACF is driven by the municipality responsible for the social service and the GP is employed by the county with responsibility of the health care.

*“I hate the organisational barriers to actually being more efficient there. I think negotiating with the nursing home to get things done is extremely frustrating.” (AusGP6)*

*“The paperwork from the municipality is often not summarized, it is time demanding work, getting it all together, and I don’t have time for that.” (SweGP4)*

##### The value of teamwork

GPs in both settings valued good leadership and skills in ACF nursing staff. Continuity of care and clear dialogue between GP and RN was perceived as a facilitator of deprescribing practice. The Swedish GPs, each only committed to one ACF, engaged regularly and effectively with the ACF’s RN. Australian GPs also placed importance on a having a good working relationship with ACF staff.

*“Well, I cooperate with the nurse named X, and she is very good and that makes my work much easier and more pleasant. We can have a good dialogue and she… I feel that she has a good clinical sense and good intentions for the patient’s well-being.” (SweGP11)*

*“I’ve got two or three nursing home staff who are fantastic. So I just don’t want make this out that they’re all terrible and that’s the reason I’m still there. If they leave I’m out of there. There’s no way I am staying.” (AusGP4)*

##### Financial incentives

A major difference between the two countries emerged in the financial arrangements for reimbursement of ACF-associated patient care. Australian GPs expressed dissatisfaction with the financial incentive for working in aged care, and felt that this was negatively influencing younger doctors’ participation in ACF work.

“*Because they [young doctors] don’t get paid enough.” (AusGP3)*

The Swedish GPs, all employed by the government, did not mention any financial factors influencing their work in ACFs or their decision to deprescribe. A government-led initiative that financially incentivises deprescribing is currently functioning in Sweden, however this does not directly affect individual GPs who work as fixed-salary employees.

#### Skills and abilities

##### Quality of human resources

There was dissatisfaction of GPs from both countries with the skill level of ACF nursing staff, which they perceived as preventing appropriate deprescribing. The GPs mentioned both level of interest and level of education of both nursing and managerial staff as being barriers for effective medication management.

*“There is a need for better educated people who run the ACFs” (SweGP10)*

*“But most [nursing staff] are very under skilled, very unintelligent and not able to make any decisions for themselves.” (AusGP1)*

Although there was some contradictory feelings about this within the Australian GP group.

“*And so – and some of the RNs are very skilled. But some of the RNs are hiding in the shadows, I reckon…* “*(AusGP6)*

##### Quantity of human resources

Doctors in Sweden also expressed the need for more staff to be able to carry out a better quality of prescribing and deprescribing practice.

*“It is frustrating with the lack of nursing staff, of course. But I can’t take responsibility for the municiptality’s employment system, it is all political.” (SweGP2)*

## Discussion

Our study has identified that deprescribing by GPs in ACFs is a complex process and that there are numerous barriers to medication reduction for aged care residents. GPs are not familiar with the evidence of the benefits of deprescribing, and this may be partially due to the lack of deprescribing trials involving participation of the frail elderly. Whilst the harms of polypharmacy are well known, most of the supporting data is observational and therefore may not be sufficient to change clinical practice, particularly in a world dominated by the marketing of medication for younger people. Despite the lacking evidence base, there is still an important role for education and continued professional development to support any deprescribing initiative.

**Fig. 2:**
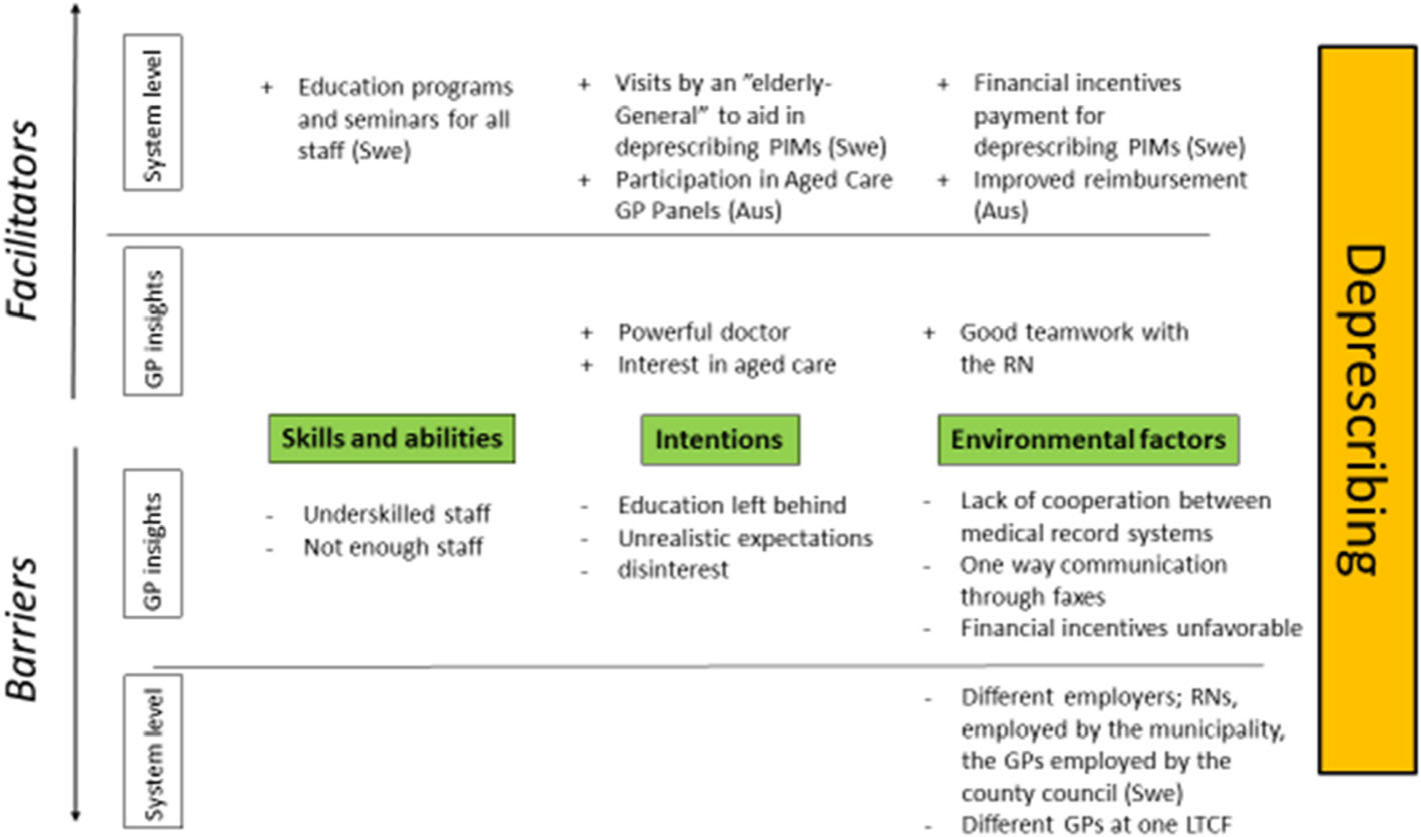
Barriers and facilitators to a General Practitioner’s participation in deprescribing for residents of aged care facilities

In Anderson et al’s review from 2014 [23] barriers and enablers to deprescribe have been summarised as a multitude of highly interdependent factors both being intrinsic to the prescriber (eg beliefs, attitudes, knowledge, skills and behaviour) and being extrinsic to the prescriber (eg. patient, work setting, health care system and cultural factors). This is also seen in our study shown in Fig. 2. Intrinsic factors as GPs attitudes seem to be very much dependent on extrinsic factors as funding for the Australian GPs. Intrinsic factors as skills and abilities for deprescribing being expressed in both countries to be hampered by the low quality in knowledge and too low numbers of ACF staff.

The attitude and belief within the ACF (of staff, relatives and residents) where medication was seen as a common solution, was considered unrealistic by GPs in both countries and there was a confidence in the doctor as a problem solver of issues that are not only medical. According to the Swedish GPs, the goal of medication management for aged care residents was to achieve a good quality of life, promoting as little medications as possible. The Australian GPs were less clear with the purpose of their commitment to aged care, with the low financial reimbursement associated with providing care for ACF residents dominating as a negative influence. Among the Australian GPs, environmental factors were evident as barriers to effective deprescribing, particularly concern over administrative burden and lack of financial incentive. GPs of both countries worried about the shortage of staff at the ACF and their lack of medication knowledge, and expressed that this limited the possibilities of deprescribing.

The systems of financial incentive for GPs working in aged care are different in the two countries. Participating Swedish GPs did not mention monetary incentives when discussing their experience with aged care services, as their work at the ACF does not influence their overall income or practice. Although there are incentives and payment for ceasing PIMS as well as extra payment for visits with multi-morbid elderly, this is provided at a system level and does not affect the individual GP’s monthly income or practice. The present funding model for GP services in Australian ACFs is less supportive of the time-commitment required to achieve meaningful medication review. Subsequently GPs appear to preference maintaining a resident’s medication list over initiating change. These findings suggest need for funding reform to better support the time and resource requirements of deprescribing within Australian ACFs, and incentivise Australian GP involvement and specialisation in aged care.

Another important difference between the two settings studied is the model of service provision: Swedish GPs are individually responsible for the care of all residents of one ACF; Australian GPs may have small patient groups across a number of different ACFs. This structural difference may have some influence on the diverging attitudes of the GPs. The Swedish model of one GP per ACF seems to favour deprescribing by promoting a closer relationship between the RN and GP and enabling clear communication around adverse medication effects. The aim of enabling good quality of life in its latter stages supported by the Swedish GPs which may be informed by the overall picture the GP gets when taking care of all residents at the ACF. The Australian GP only responsible of a couple of residents may instead have the approach of similar to overall general practice.

A systematic review of randomized clinical trials revealed that interventions using educational outreach, on-site education given alone or as part of an intervention package and pharmacist medication review may under certain circumstances reduce inappropriate drug use, but the evidence is still limited [24] and further interventions studies are needed with multidisciplinary approach.

### Strengths and limitations

It is a strength that this study has brought together evidence from GPs in different settings of the factors influencing deprescribing for AFC residents. It is important to emphasize that the results of our study are not representative of all ACFs in either setting, yet provide an illustration of factors influencing GP deprescribing for residents within the context of each setting. Limitations of participant selection have been previously described [14, 15].

When participating in an interview study, the GPs had to take time off from work. This could lead to a possibility that we may have missed the GPs that did not find the work with elderly interesting and therefore did not want to spend extra time on interviews. This may have been reflected in our study giving a more positive picture of the work at ACFs. Still, we believe that we have got more information from GPs interested in the subject than we would have got from interviewing GPs that were not interested in the task.

The analysis of the results in this study was conducted by the research team consisting of multiple professions. In light of the fact of preconceptions of the first author as a GP, who is involved in other NH studies, the co-researchers (also physicians, nurse, public health researcher) viewed the material in every step of the process of analysing data to supplement each other’s statements and interpretations in order to achieve trustworthiness [25] and analyst triangulation. In this study we synthesised a qualitative study of a sample of GPs from the south of Sweden and a qualitative study of a sample of GPs in NSW, Australia, given that the results are best interpreted in similar settings, but not necessarily transferable to other countries where ACFs may have a different structure. The interviewed GPs were of differing ages and had diverse types of experience which increased the transferability [26].

## Conclusion

Despite the high prevalence of polypharmacy along with the known risks of adverse drug effects in frail elderly, studies on deprescribing in this age group are limited but has showed positive effects in the frail elderly when drugs are withdrawn [27]. As the most vulnerable and care needing part of the elderly population reside in ACFs, the ACF population could be considered as frail and the results applicable in an ACF population.

In this study we show that the GPs’ behaviour of deprescribing in two different countries is much dependent on the larger health care system. The shortage of ACF staff is a barrier for adequate deprescribing practice. There is a need for more education to both GPs and ACF staff as well as better cooperation between the different health care systems and appropriate monetary incentives for elderly care to achieve better conditions for deprescribing practice.

## Abbreviations

ACF: Advanced Care Facilities
GP: General Practitioner
IMBP: Integrative Model of Behaviour Prediction
PHC: Primary health care
PIM: Potentially inappropriate medication
RN: Registered Nurse
